# Self-assembly study of type I collagen extracted from male Wistar Hannover rat tail tendons

**DOI:** 10.1186/s40824-020-00197-0

**Published:** 2020-11-23

**Authors:** Jeimmy González-Masís, Jorge M. Cubero-Sesin, Simón Guerrero, Sara González-Camacho, Yendry Regina Corrales-Ureña, Carlos Redondo-Gómez, José Roberto Vega-Baudrit, Rodolfo J. Gonzalez-Paz

**Affiliations:** 1 Escuela de Ciencia e Ingeniería de los Materiales, Instituto Tecnológico de Costa Rica, Cartago, 159-7050 Costa Rica; 2 Instituto de Investigación Interdisciplinar en Ciencias Biomedicas SEK (I3CBSEK), Facultad de Ciencias de la Salud, Universidad SEK, Fernando Manterola 0789, 7500000 Santiago, Chile; 3 Biological Assays Laboratory (LEBi), Universidad de Costa Rica, San Pedro de Montes de Oca, San José, Costa Rica; 4 National Nanotechnology Laboratory, National Center for High Technology (LANOTEC-CeNAT-CONARE), 1174-1200, Pavas, San José, Costa Rica; 5 National University of Costa Rica, UNA, 86-3000 San José, Heredia Costa Rica

**Keywords:** Protein aggregation, Fibrillogenic, Isoelectric point, Denatured protein, Regenerative medicine

## Abstract

**Background:**

Collagen, the most abundant protein in the animal kingdom, represents a promising biomaterial for regenerative medicine applications due to its structural diversity and self-assembling complexity. Despite collagen’s widely known structural and functional features, the thermodynamics behind its fibrillogenic self-assembling process is still to be fully understood. In this work we report on a series of spectroscopic, mechanical, morphological and thermodynamic characterizations of high purity type I collagen (with a D-pattern of 65 nm) extracted from Wistar Hannover rat tail. Our herein reported results can be of help to elucidate differences in self-assembly states of proteins using ITC to improve the design of energy responsive and dynamic materials for applications in tissue engineering and regenerative medicine.

**Methods:**

Herein we report the systematic study on the self-assembling fibrillogenesis mechanism of type I collagen, we provide morphological and thermodynamic evidence associated to different self-assembly events using ITC titrations. We provide thorough characterization of the effect of pH, effect of salts and protein conformation on self-assembled collagen samples via several complementary biophysical techniques, including circular dichroism (CD), Fourier Transform infrared spectroscopy (FTIR), differential scanning calorimetry (DSC), atomic force microscopy (AFM), scanning electron microscopy (SEM), dynamic mechanical thermal analysis (DMTA) and thermogravimetric analysis (TGA).

**Results:**

Emphasis was made on the use of isothermal titration calorimetry (ITC) for the thermodynamic monitoring of fibrillogenesis stages of the protein. An overall self-assembly enthalpy value of 3.27 ± 0.85 J/mol was found. Different stages of the self-assembly mechanism were identified, initial stages take place at pH values lower than the protein isoelectric point (pI), however, higher energy release events were recorded at collagen’s pI. Denatured collagen employed as a control exhibited higher energy absorption at its pI, suggesting different energy exchange mechanisms as a consequence of different aggregation routes.

**Graphical abstract:**

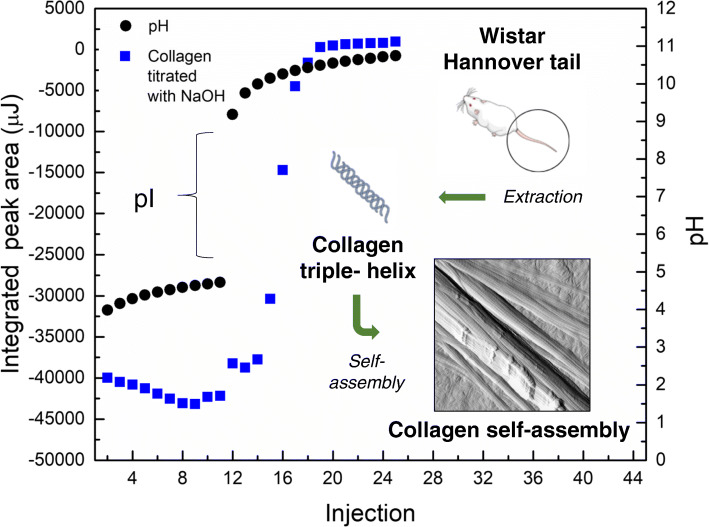

## Background

Collagen is one of the most important structural proteins, accounting for up to one quarter of protein biomass in mammals [1]. Collagen is highly abundant in animal extracellular matrixes (ECMs), and carries out not only structural functions, but acts as carrier of biological cues useful to guide cell attachment, patterning and structuring of tissues [2]. Collagen can be used not only as a scaffolding matrix for tissue engineering applications with increasing complexity [3], but also as a coating of non-biological surfaces such as polymers to improve and ensure biocompatibility of the latter [4].

Based on the shape of the fibrils and their morphological arrangements, 25 subtypes of collagen have been identified [5]. Type I, II and III collagen are characterized by their fibrillary nature, whereas type IV collagen is amorphous [6]. Type I collagen is composed of helical domains with 338 repetitions of the short motif (X-Y-Gly; wheTre X and Y usually correspond to proline and hydroxyproline) which are displayed at protein N-terminus, whereas the C-terminus presents non-helical domains [5, 7].

Importantly, there is a great demand for type I collagen, since it is the principal proteinaceous constituent of tendons, skin, ligaments and other bone tissues. Rat tail tendons are a suitable collagen source, as the lateral and transverse complex of interchain lysine-derived aldimine crosslinks get easily hydrolized under acidic conditions [8] rendering high purity collagen [9, 10]. Previously, Xiong et al. characterized and elucidated the collagen sequence from Wistar Hannover Rat tail tendons [2]. However, this previous study did not focus on the morphological and thermodynamically assembly characterization.

Collagen’s microstructure is pivotal to a number of biological events, as it helps to determine ECM cues available for cellular signaling [11]. Thus, the ways in which this structure can be modified will become relevant to direct cell response in collagen-based self-assembled materials [12]. The understanding of molecular self-assembly is a crucial factor for the fabrication of nanodevices for bioelectronics, biological actuators, artificial muscles, molecular machines, sensors, and medicine in general [13].

The self-assembly process of collagen has been previously explored [4], for instance, it has been shown that in vitro fibrillogenesis of triple-helical collagen can be controlled through pH adjustments of acid solutions which are brought to a neutral pH [5], likewise, it has been shown that adjustments in ionic strength can drive collagen fibrillary self-assembly process [4].

Calorimetric techniques represent suitable tools to monitor structural changes and biologically relevant events in protein conformation and self-assembly [14]. Isothermal titration calorimetry (ITC) is particularly useful in protein-related studies to address binding of small ligands, protein-protein interactions, drug targeting, supramolecular aggregation, and changes in enzymatic activity and inhibition, among others [15–18]. The enthalpy change of an interaction can be calculated using the raw ITC signal [14]. Interestingly, few studies have focused on the multi-step self-assembly process using ITC, for instance, Lakshminarayanan and co-workers studied the self-assembly of the protein Amelogenin and focused on studying the thermodynamic driving forces guiding supramolecular self-assembly through dilution experiments via ITC [19].

Herein we report the systematic study on the self-assembling fibrillogenic mechanism of type I collagen from rat tail tendons.

## Materials and methods

### Type I collagen extraction

Tendons were extracted from Wistar Hannover male specimens, provided by the Laboratorio de Ensayos Biológicos (LEBi, Universidad de Costa Rica, UCR). Approximately 1 g of the tendon was stirred in 200 ml of 3 % (v/v) acetic acid solution for 24 h at 4 °C. The resulting solution was filtered at room temperature with gauze and centrifuged at 4500 rpm for 30 min. The supernatant was lyophilized at 1.3 mbar, − 20 °C, for 24 h (Martin Christ beta 1–8 LSC, Osterode am Harz, Germany). In-house extracted samples were compared against commercial type I collagen from cattle bovine purchased from Thermo Fisher Scientific (catalog number A1064401, Thermo Fisher Scientific).

### Type I collagen gelation

Lyophilized collagen was dissolved in 3% (v/v) acetic acid solution while adjusting the pH to 7.47 using 1 mol/L NaOH. The solution was incubated overnight at 4 °C for gelation. The resulting gel was centrifuged and washed three times with ultrapure water (18 MΩ/cm), dialyzed with a Spectra/Por 3 membrane (32 mm diameter, 3.2 ml/cm volume), and stirred at 4 °C for over 4 days (solvent exchange took place every 2 h). For non-dialyzed collagen, these steps were omitted. Whenever collagen dry films were needed, the gel was dried out in an oven at 45 °C for 4 days.

### Collagen characterization

#### Circular dichroism (CD)

CD spectra were recorded using a J-815 spectropolarimeter (Jasco Corporation, Japan) at 20 °C in a 10 mm path length cuvette in the far UV region (190–250 nm), with signal averaging over 3 s per 0.5 nm interval at a concentration of 0.2 mM. Two repeated scans were obtained, and the baseline spectra was subtracted from the average, and blank subtraction was performed for smoothing the spectra. CD data are expressed as molar ellipticity values [20].

Fourier-Transform infrared spectroscopy (FTIR). A Nicolet 6700 spectrophotometer equipped with an Attenuated Total Reflectance (ATR) sampling accessory was used, encompassing 4000 to 400 cm^-1^ wavenumbers with a standard resolution of 0.09 cm^-1^ and a scanning speed of 32 spectra/s.

#### Differential scanning calorimetry (DSC)

Measurements were carried out on a TA Q200 instrument, using a temperature ramp of 10 °C/min with scans over the range of 20–200 °C. Aluminum containers were used, and a typical sample mass of 5 mg.

#### Amplitude modulated atomic force microscopy (AFM)

Dry films of collagen were directly deposited on freshly cleaved mica substrates, and dried at room conditions overnight. Sample topography was analyzed in air using an AFM microscope (Asylum Research, Santa Barbara, USA), operated in tapping mode. Silicon probes (model Tap150Al-G), backside with resonance frequencies of 150 kHz and force constant of 5 N/m were used.

#### Scanning electron microscopy (SEM)

Samples were deposited on the sample holders as films and gold-coated before imaging. SEM images were obtained with a Hitachi TM-3000 tabletop microscope operating at 5 kV, using charge-up reduction (low vacuum) mode.

#### Thermogravimetric analysis (TGA)

Samples were analyzed using a Q500 TA Instruments. Samples (approx. 5 mg) were placed in standard platinum pan, and mass loss change was monitored between 50 and 1000 °C.

Dynamic Mechanical Thermal Analysis (DMTA). Thermo-mechanical properties of collagen samples were determined at 25 °C using a Q500 (TA Instruments) instrument, with a testing strain of 10%, a 65-mm gap load, a 4.5 mm clamp face and a 16.66 μm/s gap speed. Tensile specimen dimensions were 4.5–5 mm width, 27–29 mm length and 1–1.6 mm thickness.

#### Isothermal titration Calorimetry (ITC)

ITC experiments were performed with a NanoITC2G (T.A. Instruments, USA). A sodium hydroxide solution (4.6 μM in ultrapure water) was titrated over aqueous type I collagen (0.2 mg/mL in 0.33%v/v acetic acid). A volume of 950 μL of the collagen solution was loaded in the cell and titrated with 24 titrant aliquots, temperature was kept constant at 30.0 ± 0.1 °C during the experiments, and the system was continuously stirred (300 rpm) with the syringe. Blank experiments were carried out by titrating 0.33% (v/v) acetic acid solutions with the corresponding NaOH titrant. All experiments were carried out at least in triplicate.

## Results

**Fig. 1:**
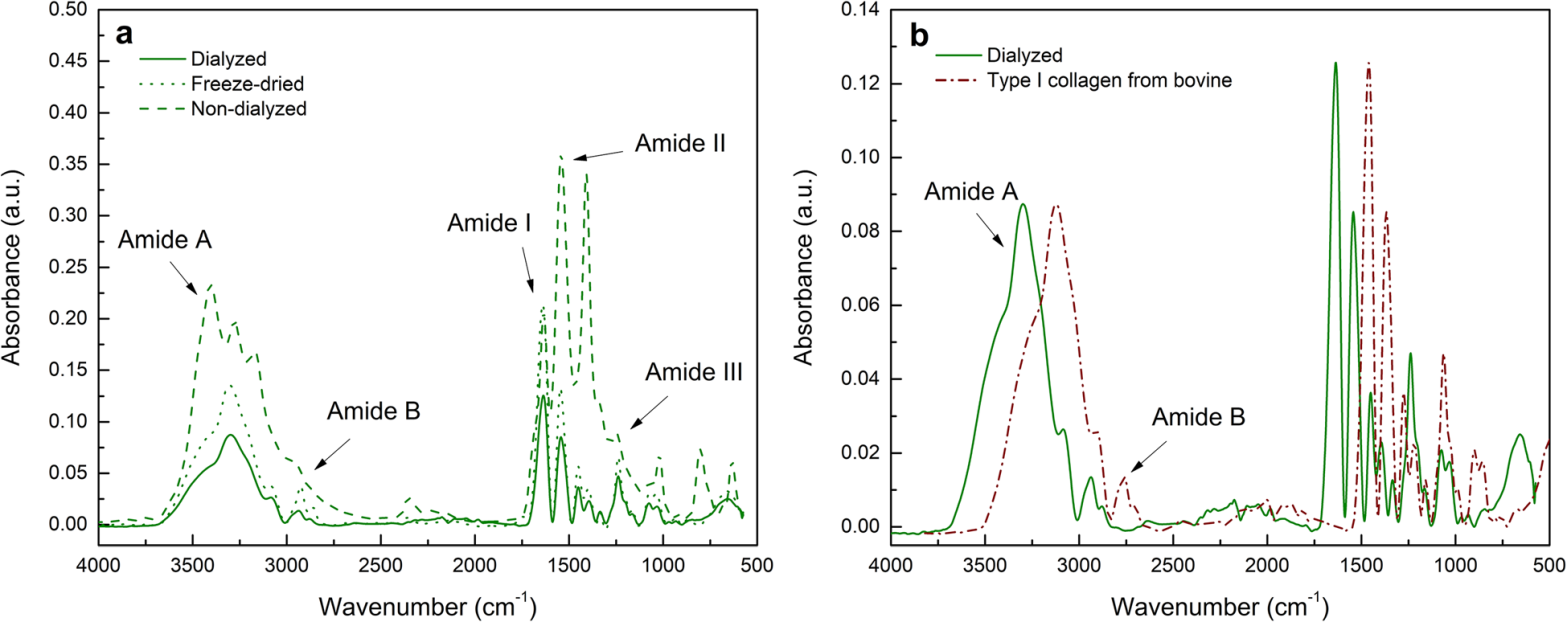
FTIR spectra corresponding to a) Extracted rat tail tendon type I collagen samples (freeze-dried, dialyzed, and non-dialyzed respectively), and b) Commercial type I bovine collagen versus dialyzed extract

Figure 1 presents FTIR spectra of extracted freeze-dried collagen samples before and after dialysis. Table 1 shows the peak centered values obtained for the Amide A, Amide B, and Amide I. Typical signals from type I collagen were observed [21], the band extending from 1640 to 1670 cm^-1^ is attributed to Amide I [21, 22], originated by the stretching vibration of the amide carbonyl group [23, 24]. The band extending from 1600 to ~ 1500 cm^-1^ is attributed to Amide II [21], it correlates the C-N stretching and the N-H bending [25]. An Amide III band was found centered at 1250 cm^-1^ [6], as a result of the bending of the N-H group and vibrational stretching of C-N [26]. Amide V bands tend to appear at a low frequency ranges from 575 to 775 cm^-1^, in this case it was found at 650 cm^-1^. This band is associated to the N-H bond’s wave motion and mainly to the CH_2_ vibrations (this band was absent in non-dialyzed collagen samples) [27].

**Table 1:** FTIR peak centered values of amide bonds (cm^-1^) and intensity ratio of Amide A (cm^-1^) /Amide I (cm^-1^)

Sample	Amide A	Amide B	Amide I	Amide A/Amide I
Type I collagen from bovine	3122	2761	1459	0.69
Dialyzed	3297	2937	1635	0.69
Non-dialyzed	3278	2967	1635	0.96
Freeze-dried	3303	2925	1639	0.63

Dialyzed samples exhibited characteristic type I collagen bands, such as Amide A around 3400 cm^-1^ (involving N-H stretching along with the hydrogen bonds) as well as an Amide B (visible near 2900 cm^-1^, involving the symmetric stretching of CH2 groups) [21].

Clear differences between dialyzed and non-dialyzed samples were found. Non-dialyzed collagen spectra showed two pronounced bands at 1540 cm^-1^ and 1410 cm^-1^, these bands indicate the presence of sodium acetate traces (representative bands at 1560 cm^-1^ and 1413 cm^-1^) produced by the neutralization of acetic acid by NaOH [28, 29], this salt is likely to remain adsorbed onto collagen self-assembled fibrils. The bands related to the C-O bond, at 1090 cm^-1^ and 800 cm^-1^, are more intense in non-dialyzed collagen samples dialysis due to presence of sodium acetate.

**Fig. 2:**
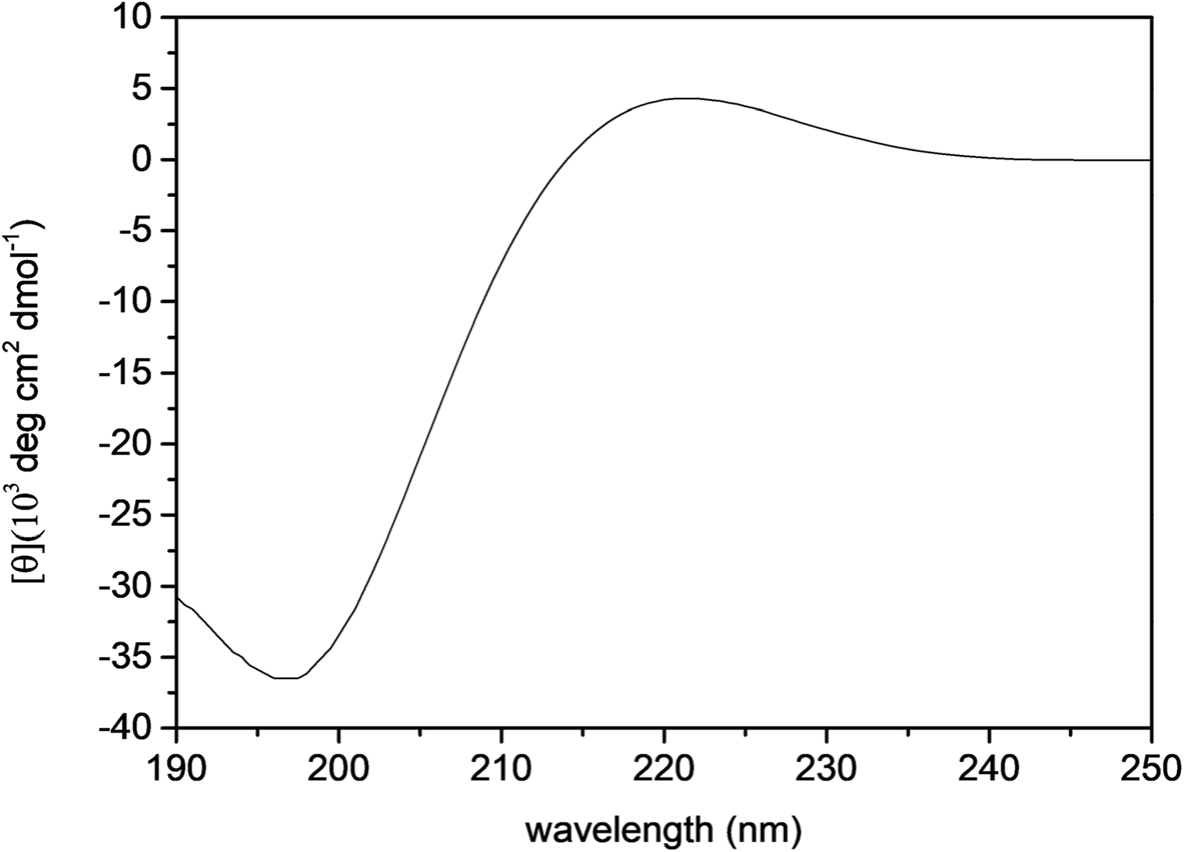
Circular dichroism (CD) spectra of a representative dialyzed rat tail tendon type I collagen sample

Circular dichroism was used for investigating the structural characterization of extracted type I collagen in solution. Figure 2 presents the CD spectra of extracted dialyzed collagen, the strong negative band centered around 200 nm [30, 31] is an indication of the canonical triple helix (TH) structure [32, 33].

Denaturation temperature of freeze-dried collagen samples was found to be 75 °C, meanwhile dialyzed and non-dialyzed samples denatured at 84 °C and 83 °C respectively.

**Fig. 3:**
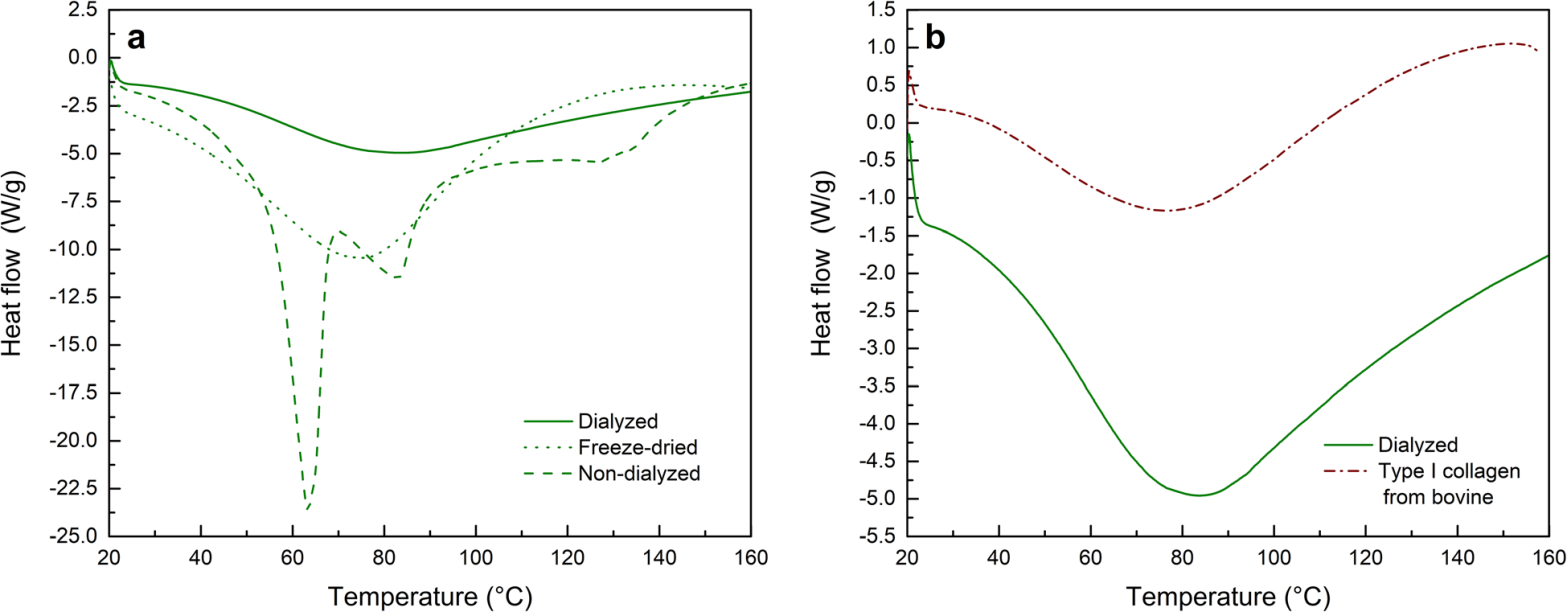
Differential scanning calorimetry (DSC) determinations on extracted and commercial collagen samples. **a** DSC thermograms of extracted rat tail tendon type I collagen at different assembly conditions: freeze-dried, self-assembled non-dialyzed, and self-assembled dialyzed. **b** DSC thermograms of type I collagen from rat tail and cattle bovine (endothermic down, exothermic up)

Figure 3 shows an endothermic denaturation temperature of 84 °C for non-dialyzed collagen, which corresponds to the unfolding of the TH structures [34], the endothermic event observed in this sample at 60 °C is associated to the melting of traces of sodium acetate hydrate [35]. This event is absent in the dialyzed collagen thermogram, indicating that dialysis treatment was effective at removing salt traces from extracted type I collagen samples.

**Fig. 4:**
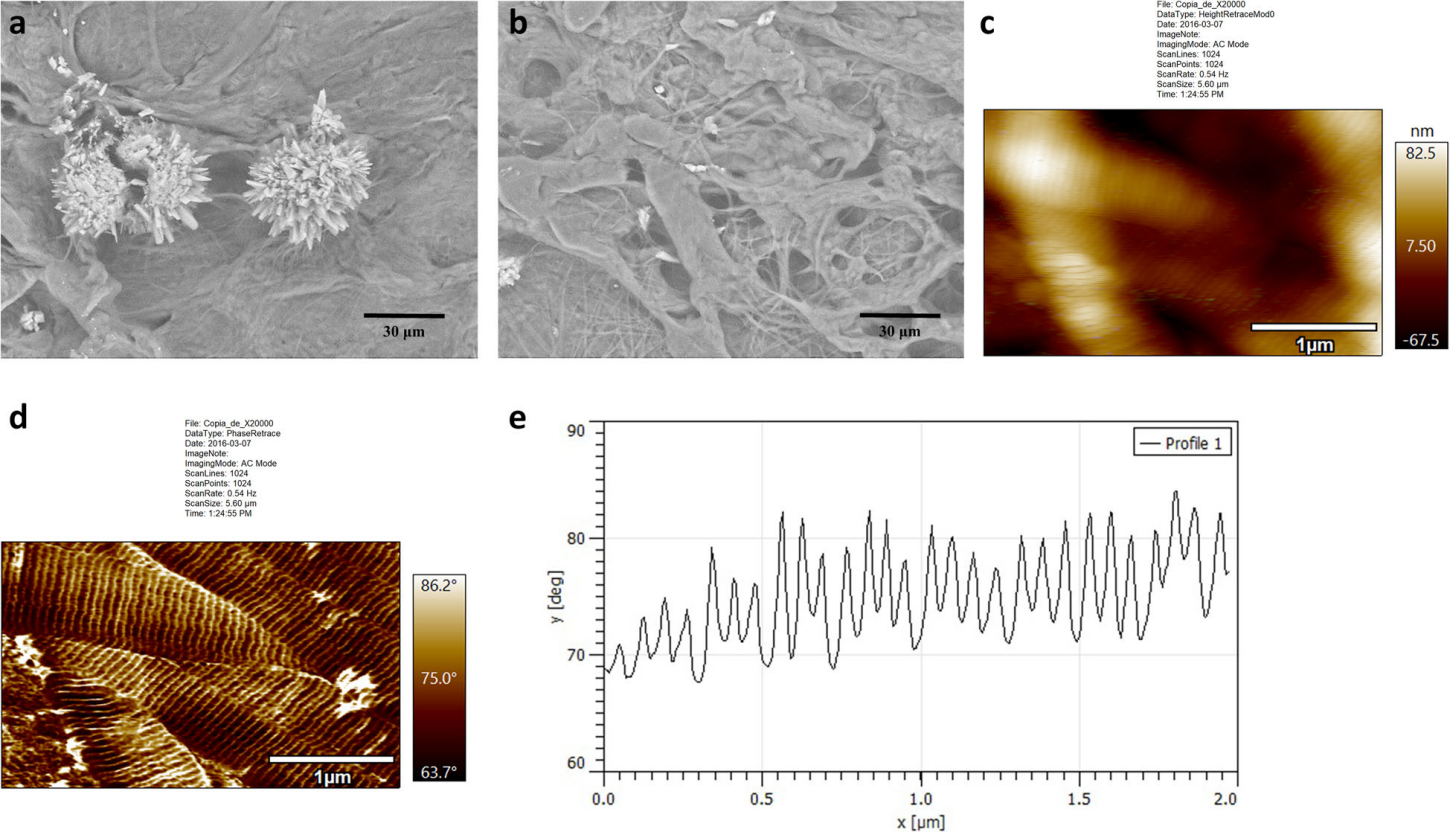
Morphological studies on self-assembled extracted collagen samples. **a** Scanning electron microscopy (SEM) images of a non-dialyzed collagen sample, and **b**) a dialyzed collagen sample. **c** Atomic force microscopy (AFM) topography image of dialyzed collagen, d) AFM phase image, and e) Line profile (as shown in panel **d** corresponding to the sample from panel c)

The morphological and mechanical characterization of self-assembled collagen was carried out using SEM and AFM. Representative scanning electron microscopy (SEM) images corresponding to non-dialyzed (Fig. 4a) and dialyzed collagen samples (Fig. 4b). Though microfibers are observed in both cases, non-dialyzed collagen clearly shows sodium acetate crystals, thus confirming that the endothermic event observed on DSC analyses at 60 °C is in fact associated with this salt. Self-assembled samples were also analyzed using atomic force microscopy (AFM) and the results are shown in Fig. 4c-e. Figure 4c shows the collagen microfibers and nanofibrils in more detail [6], the nanofibrils have a D-pattern of 65 ± 1 nm. Figure 4d shows the phase image contrast (which is associated with the voids formed between each fibril and the changes in adhesion forces between and the surface), as the AFM tip is not able to reach the bottom of these voids it interacts differently than on the fibril surface [4, 32, 33]. Each fibril diameter varies between 450 and 900 nm, which is in agreement with the reported value for the tendons fibrils up to 1 cm long and 500 nm in diameter [36].

**Fig. 5:**
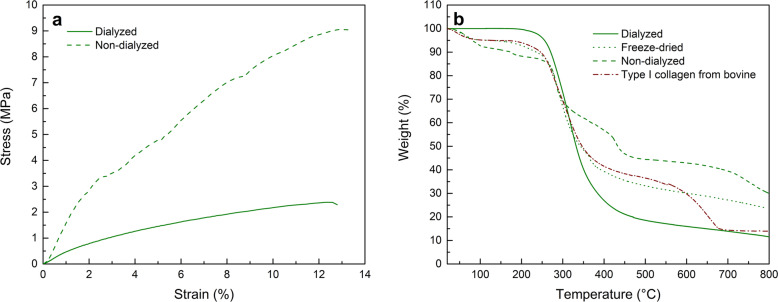
Mechanical and thermogravimetric analysis of collagen samples. **a** Stress-strain curve of dialyzed and non-dialyzed self-assembled type I collagen. **b** Thermogravimetric (TGA) thermograms of dialyzed extracted collagen and type I collagen from cattle bovine

**Fig. 6:**
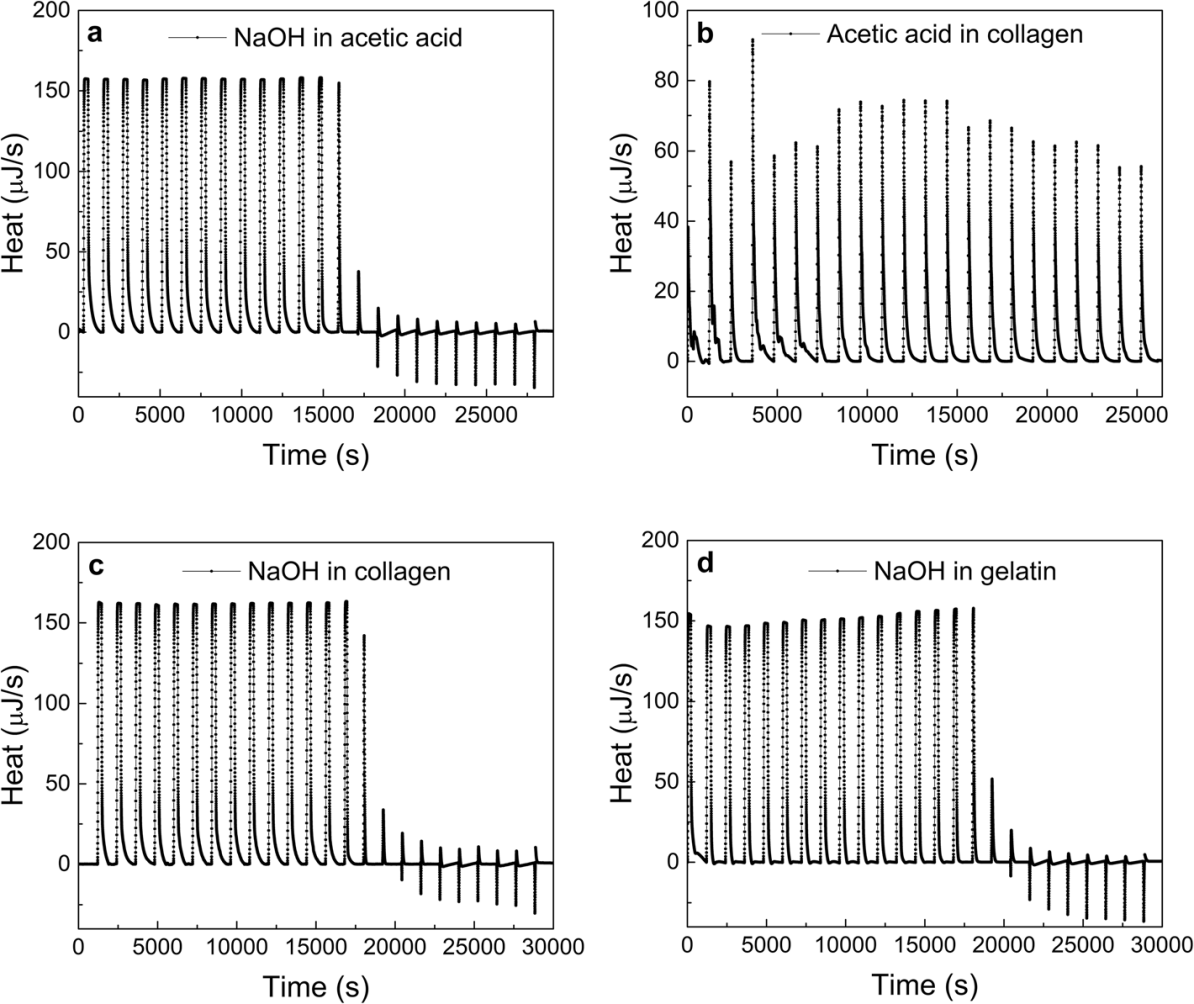
Representative ITC enthalpograms. **a** Acetic acid titration with NaOH, titration used as a control. **b** Dialyzed extracted collagen titration with acetic acid, titration used as a control. **c** Dialyzed extracted collagen titration with NaOH. **d** Denatured collagen titration with NaOH ([collagen] = 0.2 mg/mL, in 0.33% (v/v) acetic acid, [NaOH] = 4.6 μM)

Stress-strain curves of both samples are shown in Fig. 5. Non-dialyzed collagen exhibited a maximum tensile strength of 9.06 MPa, much higher than the strength obtained for the dialyzed collagen, of 2.38 MPa (Fig. 5a). Extracted samples exhibited degradation temperature ranges from 280 °C to 500 °C approximately, similar values were obtained for bovine cattle type I collagen. No high amount of impurities were detected since less than 10 wt.% degraded between 100 and 250 °C and the residues is less than 10 wt.% (Fig. 6b).

A detailed study of the collagen fibrillogenic mechanism was carried out using ITC. Initially, the neutralization reaction between acetic acid and NaOH and the collagen dissolution with acetic acid was studied. Representative ITC enthalpograms are shown in Fig. 6a and b respectively. As type I collagen was dissolved in acetic acid aqueous solutions dropwise addition of sodium hydroxide did neutralize the acid, formed a buffer system, and eventually brought up pH values above collagen’s isoelectric point (pI).

Panels c and d from Fig. 6 show ITC enthalpograms corresponding to NaOH titration of dialyzed extracted collagen and denatured collagen (gelatin), respectively. Both titrations exhibited high initial energy release due as a result of the abovementioned neutralization reaction. Cumulative additions of NaOH aliquots increase both pH and ion concentration of both systems, and both collagen titrations (panels c & d) consumed a higher amount of NaOH compared to the control acetic acid titration (panel a).

**Fig. 7:**
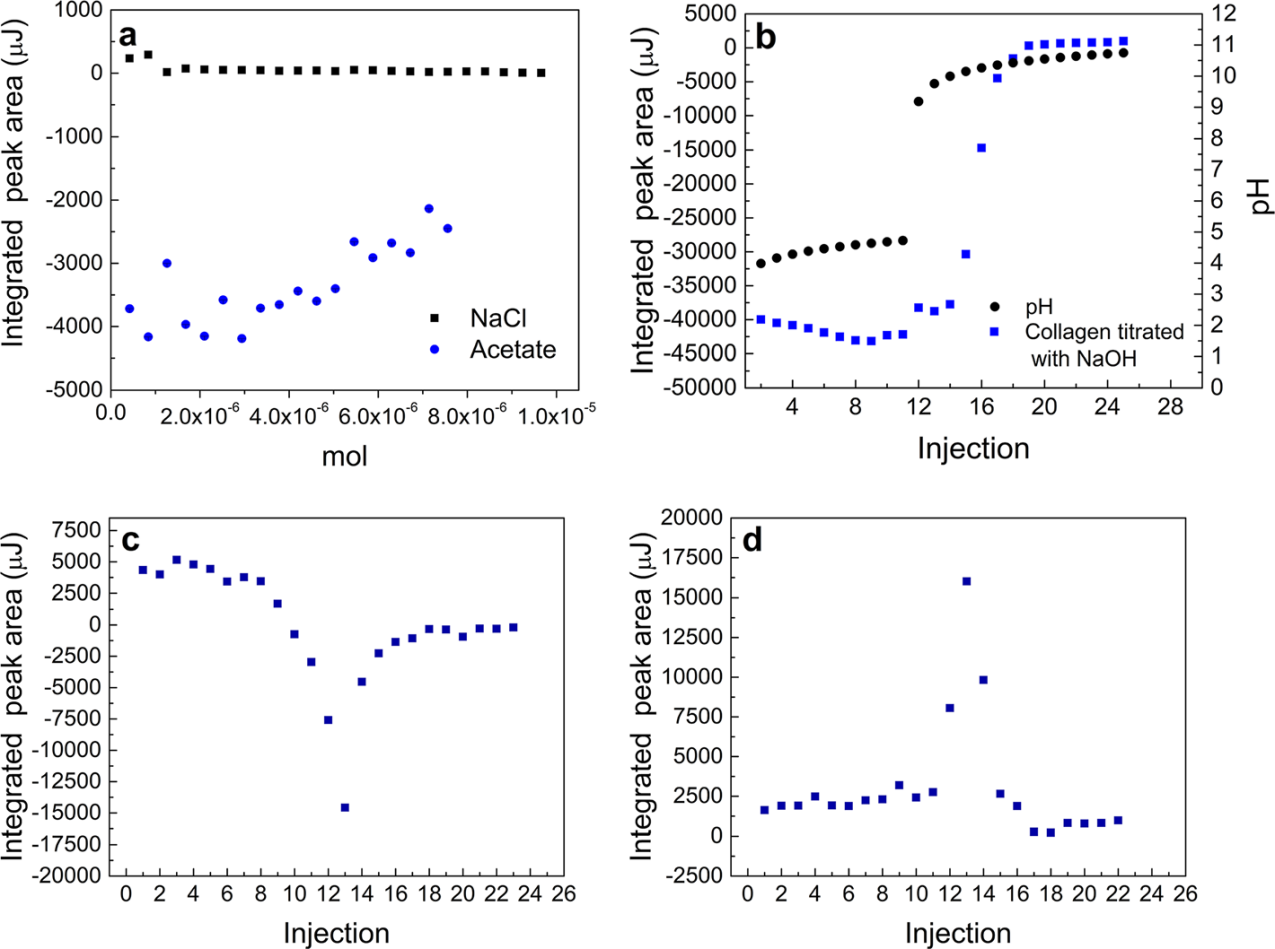
Collagen self-assembly assessment via ITC titrations. **a** Assessment of salting effect. Integrated area corresponding to the addition of NaCl (black traces) and sodium acetate (blue traces) into NaOH. **b** Integrated area corresponding to the addition of NaOH into dialyzed collagen in acetic acid (blue traces), this panel also depicts pH values measured under equivalent conditions to the ones from the respective titration. **c** Corrected area corresponding to extracted collagen in acetic acid NaOH titration. **d** Corrected area corresponding to denatured collagen in acetic acid NaOH titration ([collagen] = 0.2 mg/mL, in 0.33% (v/v ) acetic acid, [NaOH] = 4.6 μM). Integrated areas were corrected by subtracting the heat of associated to the acid-base control titration

Figure 7a shows enthalpy values corresponding to the titration of extracted type I collagen with either NaCl and sodium acetate solutions. Figure 7b depicts collagen pH changes and self-assembly progression as a function of NaOH addition. Figure 7c shows that at its isoelectric point collagen was self-assembled with an associated enthalpy value of 3.28 J/mol [4].

## Discussions

FTIR studies were carried out in order to confirm the chemical identity of extracted collagen samples before and after dialysis. The bands for the non-dialyzed are shifted in comparison to the Amide bands of dialyzed collagen. This shifting could be associated with the contributions of other proteins. The non-assembled freeze-dried collagen bands are also shifted, which might originate from conformational transitions in the self-assembled structure [6]. The differences between the peak centered values of collagen from bovine and rat tail tendons are associated with differences in the N- and C-terminus globular domains, glycosylation, and triple-helical domains [37].

Circular dichroism (CD) investigations were carried out to assess secondary structure of extracted type I collagen in solution [38, 39]. The canonical triple helix (TH) structure was confirmed [40, 41]. This TH structure is a major structural pattern observed in collagen [42], consisting of three supercoiled polyproline II Helices (ppII) formed by amino acid residues arranged in repeated X-Y-Gly triads, where X, Y are often l-proline (Pro) and 4R-hydroxy-l-proline (4R-Hyp), respectively. Hydroxylation of Pro in the Y position is essential to the fold and stabilization of TH [43], in which about 10% of the amino acids are 4R-Hyp [42].

Differential Scanning Calorimetry (DSC) studies were carried out to assess the effect of dialysis treatment on the self-assembling capacities of extracted type I collagen samples.

The results suggest that dialysis did not interfere significantly with microfibers and nanofibrils formation capacities, as both dialyzed and non-dialyzed collagen samples exhibited quite similar denaturation temperatures.

Differences between freeze-dried and self-assembled collagen samples can be rationalized in terms of supramolecular interactions between adjacent triple helixes in both cases. Self-assembled collagen microfibrillar structures are composed of several THs interacting via hydrogen bonds and forming highly organized and hierarchical microfibers. In contrast, THs present in freeze-dried collagen happen to be far more unordered, thus requiring less energy to unfold and denature as a consequence [44]. Furthermore, freezing might induce destabilization of collagen due to expansion of hydrated collagen fibrils, this might cause additional mechanical stress as well as changes in the corresponding denaturation temperature [45].

Microscopy studies were carried in order to assess any differences in fibrillogenic capacities of non-dialyzed and dialyzed collagen samples. Dialyzed collagen samples (Fig. 4b) indicate a notorious morphological organization due to the dialysis treatment. Single microfibers formed by self-assembly of nanofibrils were observed. The D-pattern matches similar values to the reported in the literature [4, 32, 33]. The microscopic and TGA analysis suggests a low amount of impurities remained in the extracted material since less than 10 wt.% degraded between 100 and 250 °C and the residues is less than 10 wt.% (Fig. 5b).

Mechanical studies were carried in order to assess any differences between non-dialyzed and dialyzed collagen samples. Both samples exhibited characteristic non-linear elastic behaviors, a response likely attributed to straightening of the TH conformation structures [46] and the alignment of both N- and C- terminus ends [47]. The higher tensile strength of the non-dialyzed collagen is most likely associated with the salt presence that confers greater rigidity to the collagen, through non-covalent salt bridges that act as cross-linking points. This in turn makes the molecules more rigid, since relaxation movements are disabled, thus increasing overall tensile strength [48]. The determination of the stress-strain relationships of individual collagen fibrils is important to understand the mechanical properties of tissues structure as well as skin, tendon, and bone.

ITC experiments were carried out in order to gain better insights of the fibrillogenic mechanism of dialyzed type I collagen samples. It was hypothesized that ITC titrations might help to identifying the factors and conditions that drive collagen macromolecular units to adopt a specific secondary conformation, which eventually lead to hierarchical formation of higher-order structures. In this fashion, type I collagen self-assembly was triggered by controlled pH increasing resulting from addition of sodium hydroxide as a titrant.

As type I collagen was dissolved in acetic acid aqueous solutions dropwise addition of sodium hydroxide did neutralize the acid, formed a buffer system, and eventually brought up pH values above collagen’s isoelectric point (pI). Collagen’s self-assembly shows dependence of pH, temperature, and protein concentration, in our ITC experiments temperature remained constant at 30.0 ± 0.1 °C, and protein concentration was higher than the minimal critical concentration for assembly [49].

Initially, control titrations of sodium hydroxide were carried out for calibration [50] as shown in Fig. 6a. Initial exothermic peaks (up) correspond to the expected neutralization reaction, while further endothermic signals can be attributed to dilution of titrant excess once the neutralization is completed. Figure 6b shows a control enthalpogram corresponding to dialyzed extracted collagen titrated with acetic acid, virtually constant exothermic peaks are an indication of the dilution of acetic acid into collagen solution with little effect on the protein conformation and self-assembly in solution.

Furthermore, higher heat release was observed on both collagen titrations compared to the control acetic acid one. This heat release difference can be rationalized in terms of a number of simultaneous molecular events taking place on collagen side chains as pH increases, including neutralization of positively charged amino acids by hydroxyl ions, resulting in changes in the electrostatic protein equilibrium, and triggering conformational changes that lead to cooperative fibrillogenesis in the last place.

Two additional experiments were carried out in order to determine whether energy release or absorption were dominated by aggregation due to a salting in or salting out effect, rather than the molecular interactions involving proton exchange leading hierarchical assembly.

NaCl values remained virtually constant along the experiment, though acetate ions had more important variations most likely due to their acid-base capacity and their well-known kosmotropic effect according to the Hoffmeister series.

These enthalpy values are both a combination of the ions dilution enthalpy and their interaction with collagen units and acetic acid, and they appear way smaller than those originated by the addition of NaOH (see Fig. 6c & d). Even though events like interactions between charged macromolecules, Debye−Hückel screening effects, and changes in water activity values are likely to take place in this scenario [51], this result suggests that energy release or absorption was driven by intermolecular interactions and is not due to collagen precipitation related to high ionic strength values.

THs of collagen can pre-assembly at pH values lower than the protein’s pI, consequently, detected heat release at lower pH values is associated to neutralization, assembly, and dissolution processes [52]. the steepest increase in corrected ∆H was observed at a pH value of 6.5, this increase is associated to higher energy release values than those obtained for the injection of acetate solutions into acetic acid (as depicted in Fig. 7a), demonstrating that this increase is not only due to dilution effects but also to changes in ionic strength. Presence of pronounced fibrillar type I collagen structures has been confirmed via AFM studies by Jiang and co-workers. This study assessed the morphology of collagen self-assembled structures at pH values ranging 2.5 to 10.5 at a fixed electrolyte concentration, finding the presence of elongated globules between pH 2.5 and 3.5, though finding fibrils in the 5.5–9.5 range [4]. To determine whether this high energy release at the isoelectric point was due to fiber self-assembly the same neutralization process was carried out using denatured collagen (gelatin) as a control. Gelatin also presented a sharp increase of a similar magnitude at its pI, though the process happened to be endothermic, a presumptive indicative of a different aggregation pathway (Fig. 7d). Type I collagen fibrillogenesis is a multi-step process driven by the increase in entropy associated with increasing molecular disorder at the water–protein interface [12]. Its nucleation growth mechanism has been reported to be driven by a polymerization reactions, starting from the monomer and made possible by the presence of covalently crosslinked oligomers [49], additionally, both hydrophobic and ionic interfibrillar interactions taking place [53], polar amino acid residues seek to establish hydrogen bonds with solvent molecules creating transient α-helices and β-sheets [54].

To form the gelatin, it is necessary to break up the secondary and higher structures of the parent protein collagen, with varying degrees of hydrolysis of the polypeptide backbone [55], from a random crosslinking of primary chains, locally twisted together, so the aggregation process can be hydrophobic effect-driven as well [56].

## Conclusions

There is no doubt that collagen-based materials are keystones as regenerative medicine constructs. In this work we provide thorough evidence that purification steps are key to define further mechanical and thermal properties of self-assembled materials based on type I collagen. Thermal analyses evidenced the importance of dialysis steps in removing salt impurities from collagen matrixes as differences in denaturation temperature were observed. In addition, it was found that salt traces can improve tensile strength of collagen-based materials.

ITC proved to be a reliable technique for studying collagen fibrillogenesis in solution state. It allowed to determine that this multi-step self-assembly process starts from pH 4.4, with an associated enthalpy of self-assembly of 3.27 ± 0.85 J/mol. Since it is so complex to understand biomolecular interactions, using ITC provided a complete heat aggregation profile of collagen fibrillogenesis. It also enabled the study of some of the factors influencing self-assembly like pH, and presence of salts. Even though ITC analysis can provide vast information regarding the fundamentals of this phenomenon there is still plenty of work to do regarding the thermodynamics and kinetics of this multi-set fibrillogenic process.

There is no doubt that consideration of collagen structure and chemistry will remain a promising area for further study. Once these details are elucidated, control over collagen self-assembly process can be exerted in order to obtaining collagenous constructs with adjustable and functional performance using different extracellular molecules that can influence its assembly at different chemical enviroments, thus determining distinctive cellular responses [14], like heart valves, ligaments and tendons, nerves, cartilage, meniscus, and blood vessels, or great use in regenerative medicine and tissue engineering applications.

## Data Availability

All data generated and analyzed during the current study are available from the corresponding author on reasonable request.

## Abbreviations

FTIR: Fourier-transform infrared spectroscopy
CD: Circular dichroism
pI: Isoelectric point
SEM: Scanning electron microscopy
AFM: Amplitude modulated atomic force microscopy (AFM).
TGA: Thermogravimetric analysis
DSC: Differential calorimetry
